# Using graph neural network and symbolic regression to model disordered systems

**DOI:** 10.1038/s41598-025-05205-8

**Published:** 2025-07-01

**Authors:** Ruoxia Chen, Mathieu Bauchy, Wei Wang, Yizhou Sun, Xiaojie Tao, Jaime Marian

**Affiliations:** 1 Physics of AmoRphous and Inorganic Solids Laboratory (PARISlab), Department of Civil and Environmental Engineering, University of California, Los Angeles, CA 90095 USA; 2 Department of Computer Science, University of California, Los Angeles, CA 90095 USA; 3 Department of Mechanical and Aerospace Engineering, University of California Los Angeles, Los Angeles, CA 90095 USA; 4 Department of Materials Science and Engineering, University of California, Los Angeles, CA 90095 USA

**Keywords:** Machine learning, Molecular dynamic simulations, Force field, Computational methods, Atomistic models, Computational science

## Abstract

The key to modeling disordered systems lies in accurately simulating atomic trajectories, typically achieved through molecular dynamic (MD) simulation. The accuracy of MD simulations depends on the precision of the interatomic potential function, which dictates the calculations of atom movements. Traditionally, deriving interatomic potential function relies on extensive prior physical knowledge and high computational cost. This study introduces a novel approach that integrates machine learning with molecular dynamic methods to provide precise interatomic potential energy calculations for disordered systems.

**Supplementary Information:**

The online version contains supplementary material available at 10.1038/s41598-025-05205-8.

## Introduction

In contrast to ordered materials, which are characterized by their periodicity and symmetrical structures, disordered materials are distinguished by their lack of long-range order and possess an inherently amorphous structure^1,2^. This characteristic makes computational simulations an highly effective tool for exploring the atomic structure of disordered materials and studying the relationship between atomic structure and material properties, tasks that are difficult to accomplish through experimental methods^3,4^.

Molecular Dynamics (MD) simulations are one of the most popular simulation techniques for simulating the movements of atoms and molecules over time. Utilizing Newton’s laws of motion, MD simulations determine the trajectories of atoms based on the atomic interaction, which are described by interatomic force fields^5,6^. Thus, the principal challenge in accurately simulating the motion and atomic structure of disordered materials lies in identifying the appropriate interatomic force field^7^. Moreover, representing the potential energy surfaces of glassy systems accurately presents significant challenges. For certain systems, analytic potential energy functions have already been established, however, these functions rely on multiple parameters that are difficult to optimize for diverse structures^4,8^. In the case of more complex structures, scientists continue to work on the development of potential energy functions^9,10^.

**Fig. 1:**
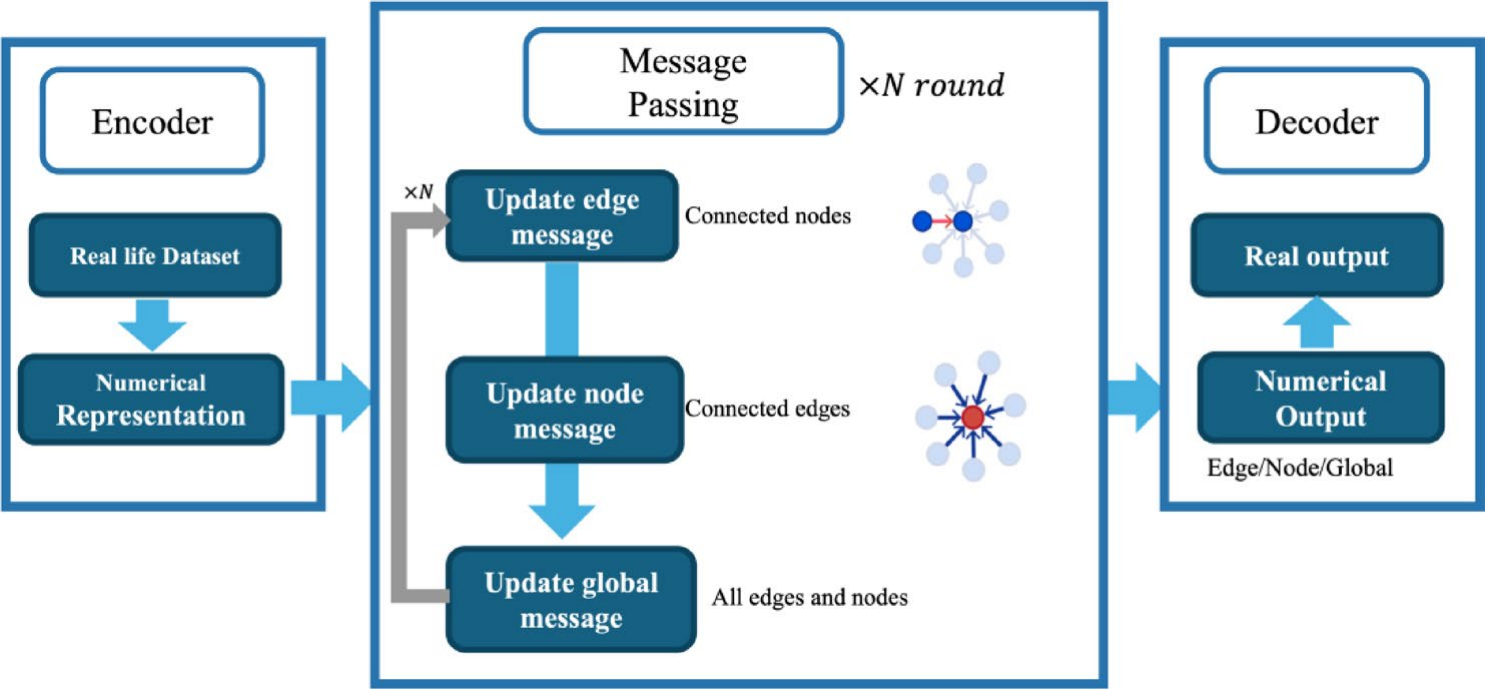
Structure of a typical gaph neural network model.

The traditional method for constructing an accurate potential energy surface involves using ab initio molecular dynamics (AIMD). AIMD employs Density Functional Theory (DFT) to calculate energies and forces based on electronic structure calculations. However, its application to large systems is limited by the substantial computational cost^11^. Given that material simulations generate a large amount of data during atomic time dynamics, machine learning (ML) algorithms have been introduced as a more efficient alternative^12^. These algorithms are excellent at processing large datasets, optimizing parameters for potential energy analytic functions^13^or directly replacing them^14^. Graph Neural Networks (GNN), one of the many ML algorithms, have increasingly captured the interest of researchers in molecular simulation due to their ability to directly capture all relevant information from molecular structure^15,16^. The GNN model takes graph-structured data as input, which contains nodes and edges that connect these nodes, mirroring the structure of the molecular system. Figure 1 shows a typical structure of a GNN model. Through a message-passing strategy, GNN models encode and update node and edge information within the input graph^17^. Unlike conventional ML models, this approach eliminates the need for human-defined features, enabling the model to autonomously identify features^18,19^.

To address the challenges associated with the ML force field, we introduce a methodology designed to transform the black-box ML force fields into analytical functions. These functions are more straightforward to interpret and can be easily integrated into molecular dynamics simulation. Our approach consists of two steps: initially, we train a GNN model to predict potential energy of a disordered system. Subsequently, we employ a Symbolic Regression (SR) model to derive an analytical function from the GNN force field. The initial dataset comprises only the system’s structure and its overall potential energy, without specific data on the pair potential energy. The GNN force field is engineered to predict not only the overall system potential energy but also the individual pair potential energies, facilitating the derivation of an analytical function for pair potential energy. SR, a model developed based on genetic programming, is capable of generating and identifying symbolic expressions that simultaneously fit the dataset without any prior assumptions^20,21^. The SR takes the system’s structure as input and uses the pair potential energies predicted by the GNN as the target output, exploring a search space consisting of a pre-specified set of mathematical building blocks that can be combined in flexible ways to generate a final symbolic expression. To validate the effectiveness of our method, we apply it to a well-studied normalized Lennard-Jones system, whose simplicity and well-understood properties facilitate a direct evaluation of the resulting functions and the accuracy of our predictions.

## Materials and methods

### Dataset

​​In our approach, two distinct datasets were employed. The first, referred to as the ‘MD dataset’, was collected during MD simulations to train and test the GNN model. Each data point in this dataset contains input values with dimensions of *R*^3^representing the three-dimensional coordinates of a system of *N* atoms, accompanied by a scalar target representing the corresponding system potential energy. The second dataset, known as the ‘GNN dataset’, was generated by processing the test MD dataset through the trained GNN model. Within the GNN dataset, each data point includes a scalar value representing the pairwise distance between atoms and a target scalar value that is the output feature from the edge model within the trained GNN for that pair of atoms. This GNN dataset was then utilized to train and test the symbolic regression model.

The MD dataset employed for training our Graph Neural Network (GNN) was obtained through molecular dynamics simulations conducted on an amorphous system in the *NVT* ensemble. To facilitate the subsequent symbolic regression step to determine the energy function, here we use a standard Lennard-Jones potential function due to its simplicity and well-studied nature:

1 $$\:{U}_{LJ}=4\epsilon\:[{\left(\sigma\:/r\right)}^{12}-{\left(\sigma\:/r\right)}^{6}]\:\:\:\:\:\:\:\:\:\:\:r<\:{r}_{c}$$

where $\:\epsilon\:$ and $\:\sigma\:$ are coefficients, each assigned a value of 1.0 to work with the normalized version of the Lennard-Jones potential. Here, $\:r$ represents the distance between two interacting atoms, and $\:{r}_{c}$, the cut off distance, fixed at 2.5 units throughout this dataset. This system consists of *N* = 128 identical atoms located within a cubic simulation box with a size of 5.44 units.

The generation of the MD dataset is an iterative process. Each iteration begins with a configuration that is randomly established. This initial state is firstly melted to a sufficiently high temperature under the *NVT* ensemble and maintained at this temperature until steady state is achieved. Subsequently, the system is quenched to a target low temperature. Once steady state is achieved at this new temperature, the atomic positions at that time step and the corresponding system potential energy are captured as a single data point. The next data point is similarly recorded in a similar manner, by quenching from the configuration of the previous data point. To eliminate spatial correlations between consecutive data points, we ensure a sufficient simulation period before recording data, allowing the system to thermalize. This period guarantees that every atom moves at least half the box size from its last recorded position, thus maintaining the independence of the data and minimizing the risk of overfitting. The cooling process within one iteration continues until the temperature of the system drops below the glass transition temperature. To ensure the dataset contains uncorrelated and comprehensive atomic structures, different target temperatures are set during the quenching steps for each iteration. This procedure is repeated until a dataset of 7,000 points is compiled.

Due to the significantly wider range of potential energy values compared to atom positions, the potential energy value within this dataset has been standardized using the Z-score normalization technique:

2 $$\:{u}^{{\prime\:}}=(u-\stackrel{-}{U})/{\sigma\:}_{U}$$

where $\:u$ and $\:{u}^{{\prime\:}}$represent the original and normalized potential energy value for each datapoint. $\:\stackrel{-}{U}\:$and $\:{\sigma\:}_{U}$ denote the mean and standard deviation of all the potential energy values across the dataset.

The GNN dataset is generated from the results of the trained GNN model. After training, a subset of the MD dataset was input into the GNN model. The outputs of the edge model, along with the corresponding atom pairs, were recorded. The pairwise distances serve as the input values, and the recorded edge output features are used as target values. Further details regarding the datasets will be discussed in the Results Sect. 3.3.1.

### Graph neural network

**Fig. 2:**
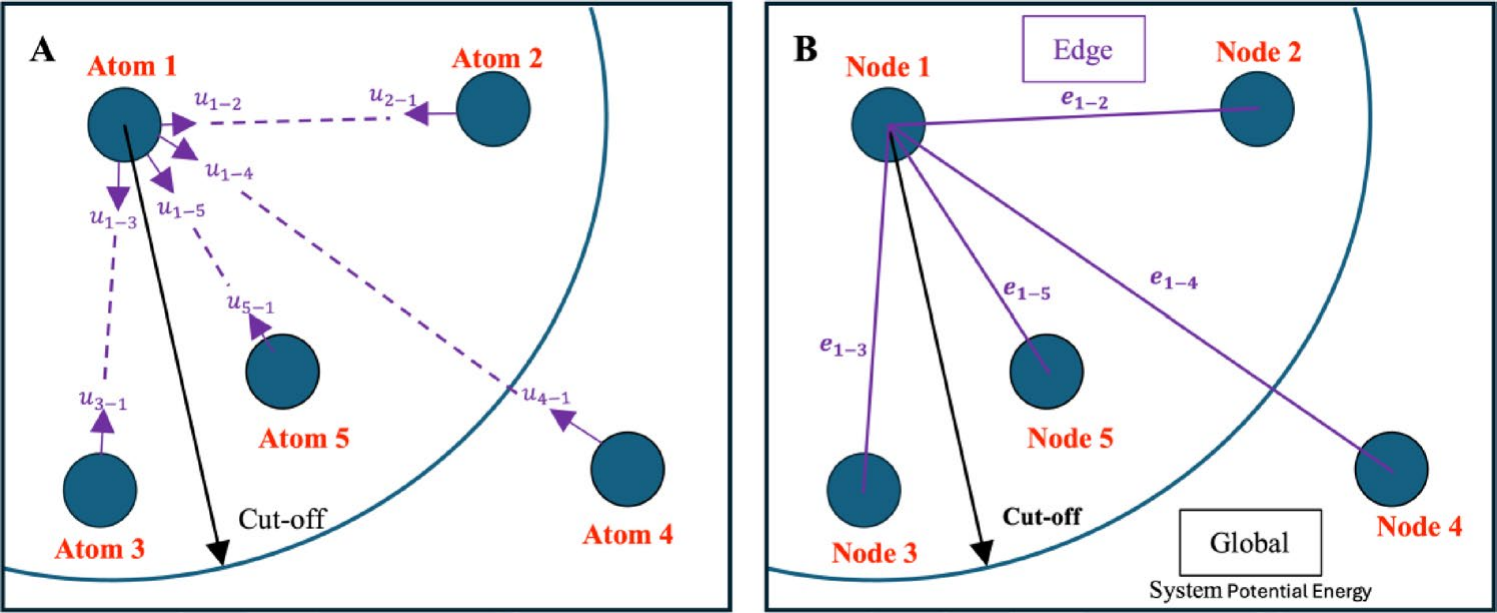
(**A**) Atomic system contains five atoms (**B**) graph representation for this system, in which each atom is represented as a node, edges describe atomic interaction and global correspond system properties, the system potential energy.

The graph neural network (GNN) model is designed specifically for handling graph-structured data, which contains features such as vertices and edges. As shown in Fig. 2, the left figure represents an atomic system, where $\:{u}_{i-j}$ denotes the potential energy caused by the interaction between atom $\:i$ to atom $\:j$. The right figure shows the graph representations for this system. In this structure, vertices, or nodes, symbolize the entities within the graph, which are atoms in our dataset. Edges denote the relationships between these entities, such as the distances between atoms. The $\:{e}_{i-j}$ in the Fig. 2B represents the edge between node $\:i$ to node $\:j$. This setup allows for the transformation of molecular dynamics simulation output structures into a graph-structured format, enabling their analysis via the GNN model.

In our dataset, each datapoint is structured as an undirected, unweighted graph comprising three elements: node, edge and global feature. Nodes are denoted as $\:{V}^{\left(G\right)}\in\:{R}^{n}$, with each node featuring attributes describing one atom. Given that the normalized Lennard-Jones system contains only one type of atom, the node attribute for atom species is uniformly set to 1. Edges in the graph symbolize the relationship between atom pairs, represented as $\:{E}^{\left(G\right)}\in\:{R}^{n\times\:n\times\:3}$. These edges are characterized by attributes indicating pairwise spatial coordinate differences. When the spatial distance between two atoms is less than the cutoff distance, the edge attribute is defined as:$\:{e}_{ij}=\left[\varDelta\:{x}_{ij},\:\:\varDelta\:{y}_{ij},\:\varDelta\:{z}_{ij}\right]=[{x}_{i}-{x}_{j},{y}_{i}-{y}_{j},{z}_{i}-{z}_{j}]$. For distances exceeding the cutoff distance, we assume there is no relationship between these two atoms, setting: $\:{e}_{ij}=\left[\text{0,0},0\right]$. The cutoff distance is fixed at half the length of the simulation box. The target of the graph, $\:{U}^{\left(G\right)}\in\:{R}^{n}$, is the system’s potential energy. Thus, each graph can be represented as $\:G({V}^{\left(G\right)},{E}^{\left(G\right)},{U}^{\left(G\right)})$. The output of the GNN model for each graph is the global output $\:{Y}^{\left(G\right)}\in\:{R}^{n}$, which aims to closely match the value of the corresponding target. Consequently, the cost function for this GNN model is established as the Mean Square Error (MSE) between the output $\:{Y}^{\left(G\right)}$ and target$\:\:{U}^{\left(G\right)}$.

Given the simplicity of the normalized Lennard-Jones system, the GNN model adopts a simplified message-passing neural network architecture to avoid overfitting. The GNN model updates edge, node, and global features through message propagation. The updating process starts with the edge features, which are refreshed by inputting their own values and those of adjacent node features into an edge Multi-Layer Perceptron (MLP)^22^. All edge features are updated synchronously using this edge MLP. Subsequently, the node features are updated via a node MLP, with inputs being the node’s own features and the freshly updated edge features associated with that node. All node features are updated simultaneously using this node MLP. Based on the prior knowledge that the system’s potential energy in a Lennard-Jones system is the aggregate of pairwise potential energies, the graph’s output is calculated by taking half the sum of the node features. This approach is designed to closely mimic the potential energy calculation process in molecular dynamics simulations.

As indicated in Table 1, the message-passing steps in the GNN model closely mirror the process of calculating potential energy in MD simulation. The output from the edge model, represented by the updated value of each edge feature, can be considered equivalent to the potential energy between pairs of atoms, implying that the edge model performs the same functionality as the Lennard-Jones function. Similarly, the updated node feature values are analogous to the potential for training, the output of the global model aims to match with the potential energy of the whole system. Through this process, the GNN model is capable of not only predicting the overall system potential energy but also predicted potential energy for each atom pair. This provides an opportunity to reconstruct the pair energy function.

**Table 1:** Comparative steps in GNN message passing and potential energy calculation in MD simulation.

GNN message passing steps	Potential energy calculation steps
Edge model: $\:{e}_{ij}^{{\prime\:}}={\varphi\:}^{e}({e}_{ij},{v}_{i},{v}_{j})$	Each pair: $\:{u}_{ij}=4\left[\right(1/{r}_{ij})^{12}-(1/{r}_{ij})^{6}]$
Node model: $\:{v}_{i}^{{\prime\:}}={\varphi\:}^{v}({\stackrel{-}{e}}_{i}^{{\prime\:}},{v}_{i}),\:{\stackrel{-}{e}}_{i}^{{\prime\:}}={\varSigma\:}_{j}{e}_{ij}^{{\prime\:}}$	Each atom: $\:{u}_{i}={\varSigma\:}_{j}{u}_{ij}$
Global model: $\:{Y}^{{\prime\:}}={\varphi\:}^{u}({V}^{{\prime\:}},{E}^{{\prime\:}}),$ $\:{V}^{{\prime\:}}=\{{v}_{i}^{{\prime\:}}{\}}_{i=1:N},\:{E}^{{\prime\:}}=\{{e}_{i}^{{\prime\:}}{\}}_{i=1:N}$	Whole system: $\:{U}_{Total}=\frac{1}{2}{\sum\:}_{i}^{N}{u}_{i}$

### Symbolic regression

After training the GNN, we utilize Symbolic Regression to derive an analytical expression from the trained edge MLP. Given that the Lennard-Jones function describes a two-body interaction where the total potential energy is the sum of the pairwise potential energies, it is crucial for the trained GNN to capture these pairwise interactions from total potential energy effectively and accurately. To enhance this capability, the GNN architecture was streamlined, specifically designed to replicate the total potential energy calculation steps in MD simulation. Modifications were made to both the Node MLP and Global MLP, simplifying them to perform the sum of all their inputs. This adjustment streamlined the model, enhancing its efficiency. Consequently, from the refined GNN, the output of edge MLP more precisely predicted the pairwise potential energy for each atom pair, which will be discussed in the Result Sect. 3.2. In this way, a great dataset can be collected from the GNN model, which is subsequently used to train the symbolic regression model.

The dataset is derived from the trained GNN model. After the GNN is trained with the training set, for each test datapoint entered, we capture both the input and the corresponding output from the edge MLP. Each MD datapoint includes 128 nodes, resulting in 16,384 potential edges among those nodes. According to the definition of edge feature, edges exceeding the cutoff value are assigned a feature value of zero. Therefore, our data collection focuses only on edges with non-zero features. Based on prior knowledge and to simplify the symbolic regression process, the original input, spatial coordinate difference $\:\left[{\varDelta\:x}_{ij},{\varDelta\:y}_{ij},\:\varDelta\:{z}_{ij}\right]$, is converted into a singular distance metric $\:{r}_{ij}=\sqrt{{\varDelta\:x}_{ij}^{2}+{\varDelta\:y}_{ij}^{2}+{\varDelta\:z}_{ij}^{2}}$ as input into symbolic regression. The aim of symbolic regression is discovering a function that accurately maps the input distance to the output edge features produced by the edge MLP.

The symbolic regression was conducted using the PySR^23^ package, which applies a genetic algorithm to fit the dataset obtained from the trained GNN. The PySR implements this algorithm to generate and identify optimal mathematical equations by randomly combining input variables, constant and basic operators such as addition ($\:+$), subtraction ($-$), multiplication ($\:\times\:$), division ($\div$), exponential (exp), inverse (inv), and power (pow). Each expression is evaluated and selected based on its complexity and accuracy throughout the generation and mutation process. The outcome is a list of candidate equations, from which we select the final equation based on its performance on test data and its complexity. More details are provided in the Result Sect. 3.3.

## Results

### MD dataset

**Fig. 3:**
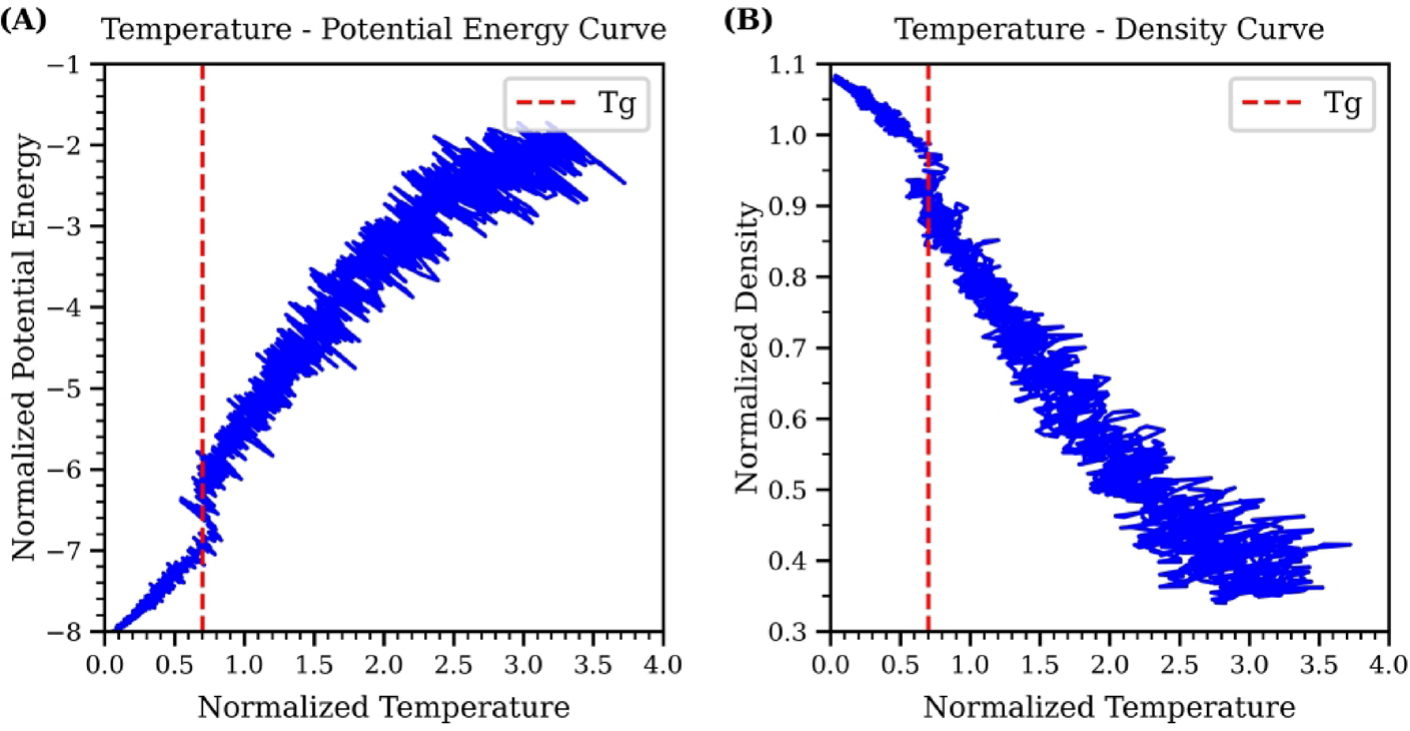
(**A**) Temperature-Potential Energy curve for the normalized Lennard-Jones system. (**B**) Temperature-Density curve. Both curves show a noticeable change in slope around the glass transition temperature of $\:{T}_{g}$= 0.7, as indicated by the red dashed line.

The MD dataset was generated by running MD simulation for the 128-atom system using the normalized Lennard-Jones potentials. First, the glass transition temperature ($\:{T}_{g}$) for this system was determined by analyzing changes in potential energy and density with respect to temperature^24^. The system was initially melted to a high temperature of 3.5 units to reach a stable state. Subsequently, the system was quenched from 3.5 units to 0.01 units with a cooling rate of $\:{10}^{-5}$ units per time step. The changes in potential energy and density as functions of temperature during the quenching process are plotted in Fig. 3. Both curves exhibit a constant change with temperature, followed by a sharp change in slope near $\:{T}_{g}$. According to the curves in Fig. 3, the changes in slope occur around a temperature of 0.7, which is identified as $\:{T}_{g}\:$for this system.

**Fig. 4:**
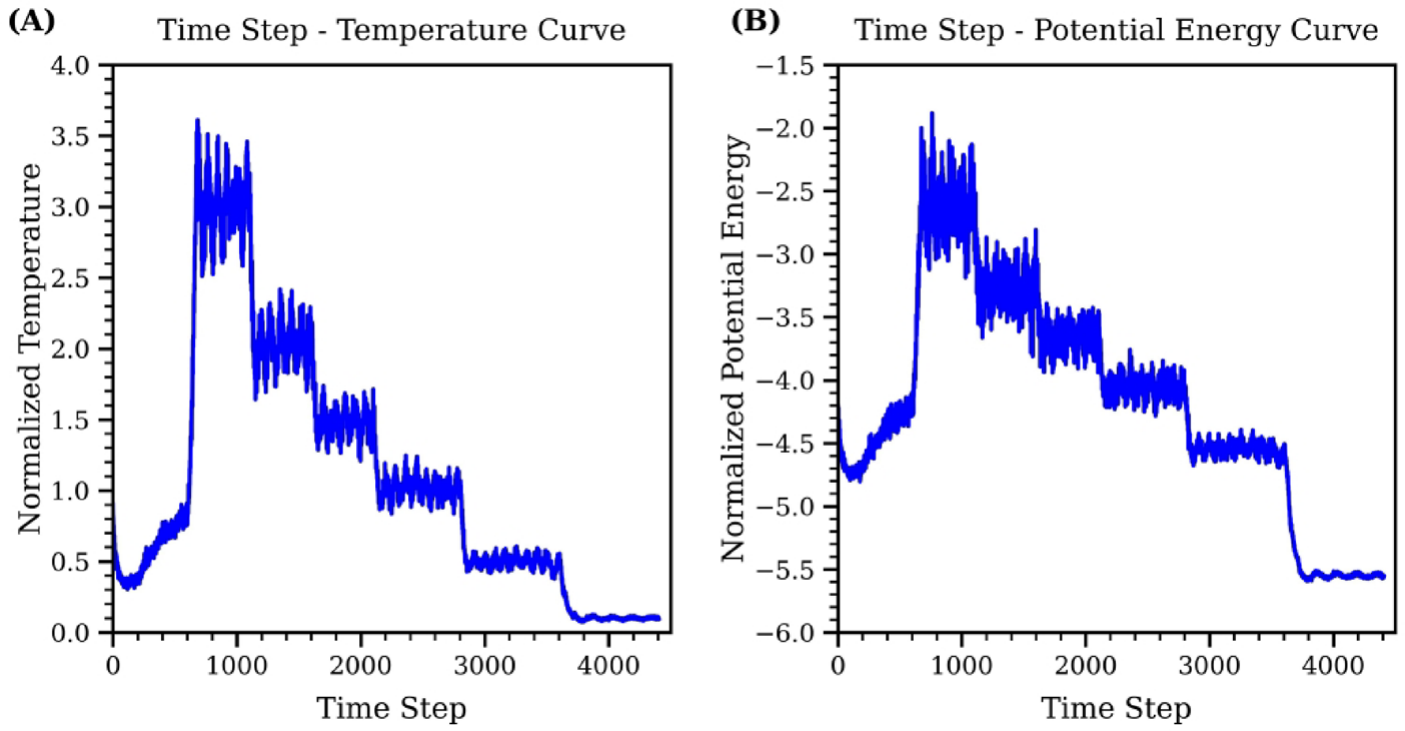
The quench process for the first iteration to collect MD dataset. (**A**) shows the change of system temperature during the simulation process and (**B**) represents the change of potential energy with respect to time steps during the quench process.

After determining the glass transition temperature for the system, the MD dataset was collected through an iterative quench process, as discussed in Sect. 2.1. Figure 4 illustrates the first iteration of the data collection process. Figure 4A displays the changes in temperature during the quench process. Starting from a random configuration, the system is melted to a high temperature of 3.0 units using the *NVT* ensemble. Once the system stabilizes at this temperature, a data point is recorded. Then, the temperature was systematically decreased from 3.0 units to a minimum value of 0.2 units, below the glass transition temperature. This temperature curve reflects the system’s response to incremental decreases in set temperature, capturing the stabilization of the system at each new temperature before further reduction. Figure 4B shows the corresponding potential energy curve for the evolution shown in Fig. 4A. The potential energy fluctuates with each temperature adjustment and then stabilizes after a few hundred-time steps. This quench process was repeated multiple times with various target temperatures until a comprehensive dataset was collected.

### GNN results

The MD dataset consists of 7,000 data points, randomly divided into training and testing set, with 70% allocated as the training set and the remaining 30% as the test set. The GNN architecture is intentionally streamlined to reflect the physical process of potential energy accumulation in molecular dynamics. The only learnable component in the network is the edge MLP, which consists of four fully connected layers with hidden sizes (8, 16, 16, 8), followed by a final output layer of size 1. The ReLU activation functions are applied after each hidden layer, with no activation applied at the final output layer. The model uses a single message-passing step (i.e., one recurrence), and no dropout is applied during training. Both the node and global updates are implemented as summation operations rather than parameterized networks, in accordance with the additive nature of the Lennard-Jones potential. The total number of trainable parameters in the GNN model is approximately 609. This minimal and physically grounded design reduces the risk of overfitting while enabling the edge model to effectively learn pairwise interactions that approximate the underlying potential energy function.

The accuracy of this model is evaluated by the root mean square error (RMSE) between the predicted global output and the system potential energy collected from MD simulation. Our model was trained using the Adam optimizer, with a learning rate of 10^− 3^ and a batch size of 64. The GNN achieved an average RMSE per atom of 0.028028 across the test set, with a standard deviation of 0.011142, based on five independent runs with different random seeds. This result indicates that the discrepancy between the predicted and actual potential energy per atom is only about 0.028028 units, corresponding to a relative error of approximately 1.36% compared to the average potential energy per atom of  -2.0542 in the MD dataset. More detail about the GNN training result can be find in Supplementary Information.

**Fig. 5:**
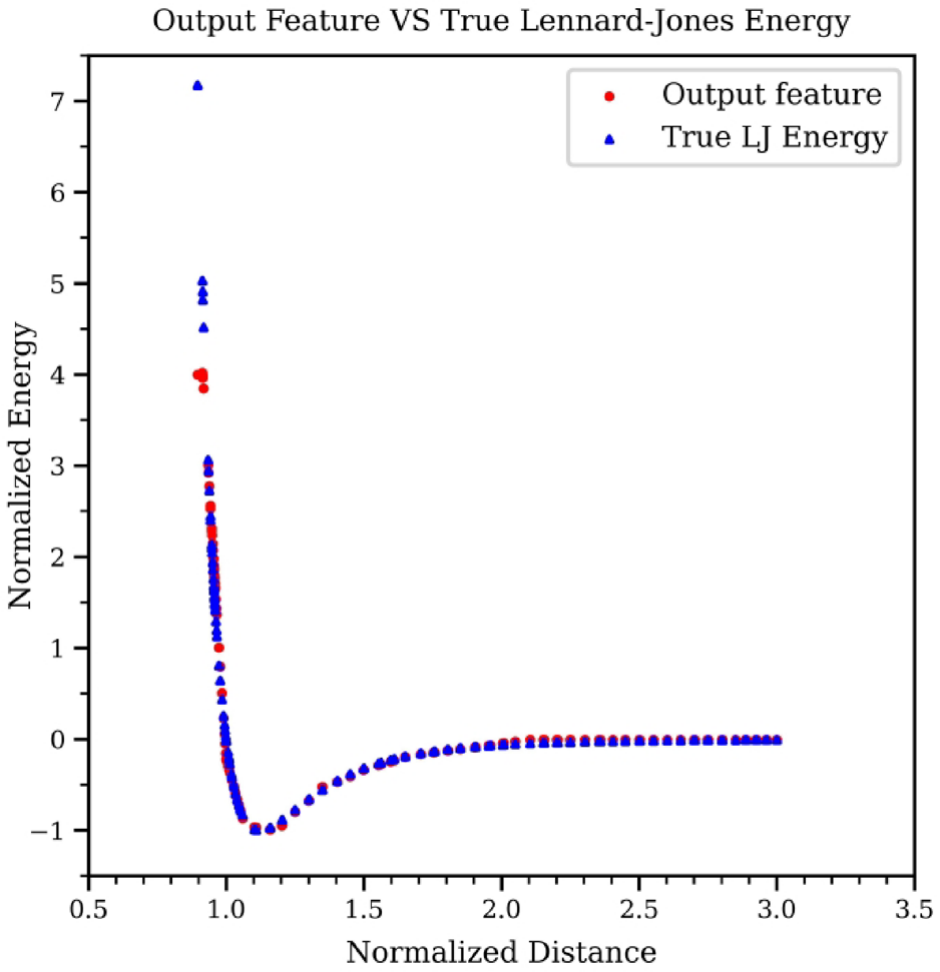
Comparison of GNN model’s edge output features against true Lennard-Jones potential energy values.

After training the GNN, the edge features output from the edge model is recorded by evaluating the test set with the one trained GNN. Only non-zero edge features are retained, corresponding to atom pairs with distance within the cutoff distance. In Fig. 5, the *x*-axis displays atom distances, while the *y*-axis represents potential energy values. This figure shows that the trends of GNN output feature values with respect to atom distances are similar to the trends between the ground truth Lennard-Jones values and atom distances. The mean square error (MSE) between the output features and the corresponding Lennard-Jones potential energy values at identical distances is 0.1365. This accuracy suggests that the edge output features effectively capture pair potential energy values. Significantly, as the node distance increases, the difference between the predicted edge output and the actual Lennard-Jones value decreases. The reduction of error indicates that the GNN model performs better at large atom distances than at small atom distances.

### Symbolic regression results

#### Dataset influence

The GNN model demonstrates high accuracy in predicting potential energy at longer atom distances, yet this accuracy decreases for compact systems. This trend indicates that the system temperature within the dataset, referring to the temperature conditions under which the data was collected during MD simulations, could influence the accuracy of GNN model, and by extension, the accuracy of the symbolic regression. The fact that temperature influences the average separation between atoms is no surprise on account of the positive thermal expansion predicted by the Lennard-Jones potentials, but the observation that it also impacts the GNN prediction accuracy is interesting. To further investigate how the average interatomic distance affects the symbolic regression’s ability to generate the desired function, we divided the test dataset used to evaluate the GNN, obtained from MD, into two subsets. These subsets are distinguished based on the temperature at which the systems were simulated, with the glass transition temperature acting as the threshold.

The test dataset collected from MD simulation, categorized by system temperatures, forms two distinct sets: the high-temperature MD dataset for temperatures above the glass transition temperature point and the low-temperature MD dataset for temperatures below it. Each subset was independently processed through the trained GNN model to extract the edge output feature, as detailed in the GNN result Sect. 3.2. Consequently, two different GNN datasets were produced that will used to train the Symbolic Regression: one representing atom pairs with their corresponding edge output features from the high-temperature MD dataset, and the other comprising atom pairs from the low-temperature MD dataset. The low-temperature dataset contains 18,758 data points, while the high-temperature dataset includes 19,820 data points. For both datasets, a random split of 70% for training and 30% for testing was applied.

**Fig. 6:**
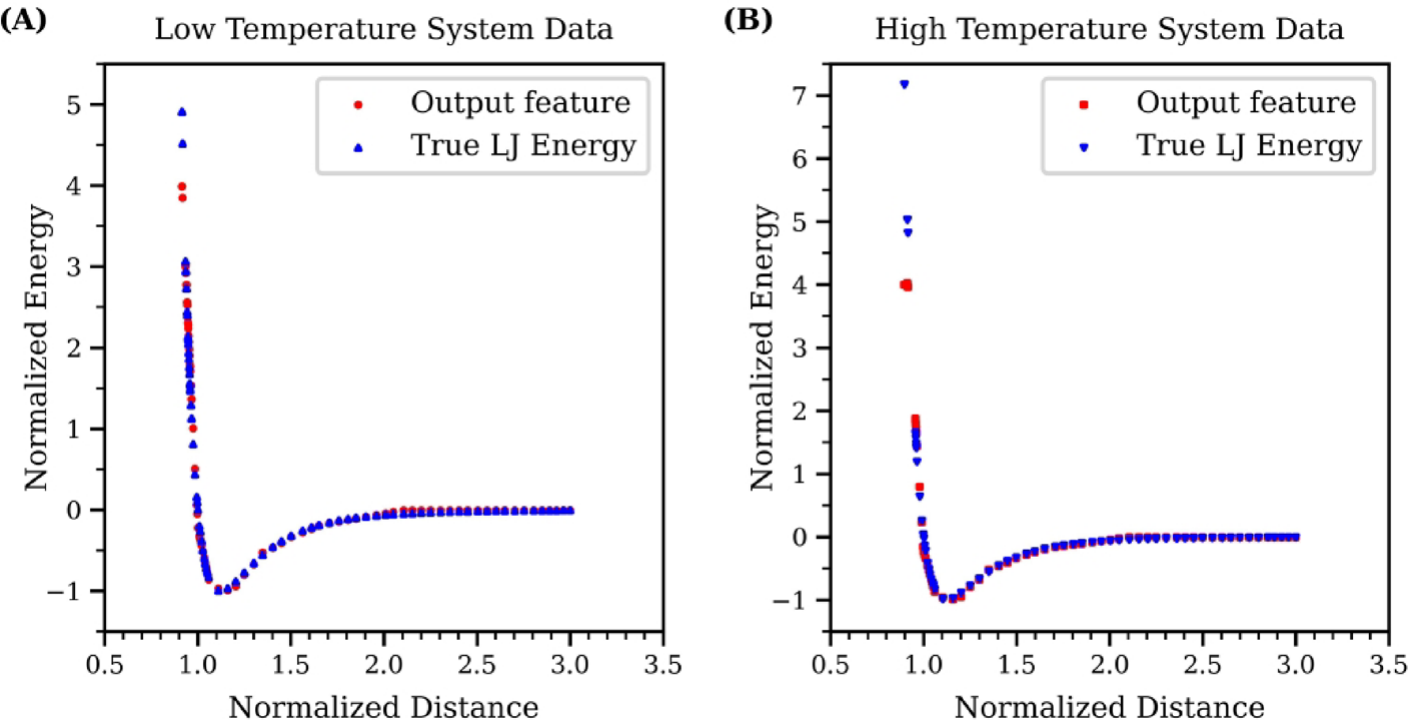
Energy-distance curves for GNN edge outputs and Lennard-Jones potential. (**A**) depicts the correlation for the low-temperature GNN dataset alongside the true Lennard-Jones potential at matching distances. (**B**) shows the same for the high-temperature GNN dataset.

Figure 6 illustrates a comparison of the edge output features from these GNN datasets against the value calculated through the ground truth Lennard-Jones function at equivalent distances. The MSE between the low-temperature GNN dataset with Lennard-Jones value is 0.00065, while the high-temperature GNN dataset has an MSE of 0.00165. In both datasets, there is a clear correlation between the predicted features and atom distances which follows the trends of Lennard-Jones potential energy function, especially when the atom distance exceeds the equilibrium lattice parameter, where the potential energy is at its lowest. A notable difference is that the low-temperature GNN dataset includes a higher number of data pairs with distances shorter than this equilibrium distance, as opposed to the high-temperature GNN dataset. This is consistent with MD simulation behavior, where low temperatures result in reduced atomic kinetic energy, hence atoms tend to be close to each other and form a compact structure similar to a solid. The impact of this difference for the results of symbolic regression will be explored later. More detail about the data distribution for these tow datasets can be find in in Supplementary Information.

#### Influence of temperature

To investigate the impact of system temperature on symbolic regression’s ability to reconstruct the normalized Lennard-Jones energy function, we utilized the previously described high and low-temperature GNN datasets. Each dataset was divided into training and a test set. Symbolic regression then generates a list of candidate equations from the training data and evaluates them with the test set. Evaluation metrics included complexity, loss, and trade-off score. Complexity is calculated by counting the number of operators, constants, and variables in an equation. Loss is measured by the mean squared error between the predictions of the equation and the target value, and the improvement considers both loss and complexity. The list of candidate equations was restricted to those with a complexity below 50, sorted in ascending order based on their complexity. The trade-off score for the *i*-th equation in this candidate list is determined as follow:

3 $$\:{Score}_{i}=-\text{l}\text{o}\text{g}({loss}_{i}-{loss}_{i-1})/({Complexity}_{i}-{Complexity}_{i-1})$$

**Fig. 7:**
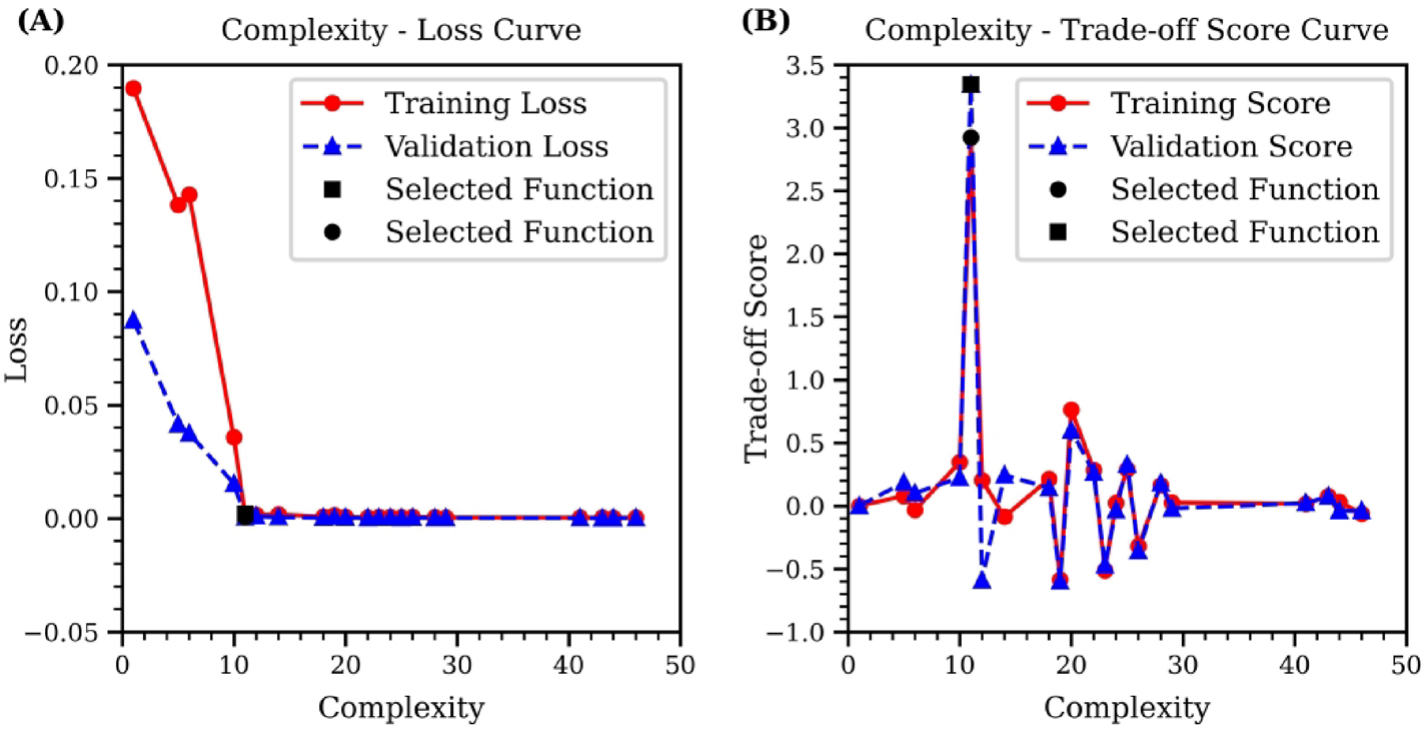
Complexity curves of symbolic regression using low-temperature GNN data. (**A**) illustrates the Complexity-Loss curve, highlighting the optimal equation at the turning point of minimal loss. (**B**) displays the Complexity-Trade-off Score curve, where the highest trade-off score indicates the selected equation that best balances simplicity with predictive accuracy.

The trade-off score shows whether an increase in complexity brings enough performance improvement, which is important for preventing overfitting. A high trade-off score helps in selecting the optimal equation that achieves the best balance between complexity and accuracy,

To choose the optimal equation from the candidate list as a result, we calculate the loss and trade-off score for each candidate based on the training and test dataset separately. Figure 7A provides the relation between complexity and loss. Initially, as complexity increases, both training and test loss decrease, reaching a plateau after a certain point. Thereafter, additional complexity fails to significantly improve equation performance. The equation positioned at this turning point is identified as the optimal choice, which achieves an optimal balance between complexity and loss to prevent overfitting while maintaining accuracy. The effectiveness of the selected equation is further verified in Fig. 7B, which displays the Complexity-Trade-off Score curve. By definition, the highest trade-off score indicates an equation that offers the best compromise between loss and complexity. The curve demonstrates that the selected equation achieves the highest trade-off score across both the training and test set. The selected function is as follows:

4 $$\:y=\:3.9584014(1-{x}^{6})/{x}^{12}$$

This equation mirrors the structure and exponents of $\:x$ in the target function, with only a slight variance in parameter’s numerical value from the target normalized Lennard-Jones function (1), which uses a parameter of 4 compared to 3.9584014 in the selected equation. This outcome demonstrates that our methodology successfully predicts the Lennard-Jones potential energy function using the low temperature dataset.

**Fig. 8:**
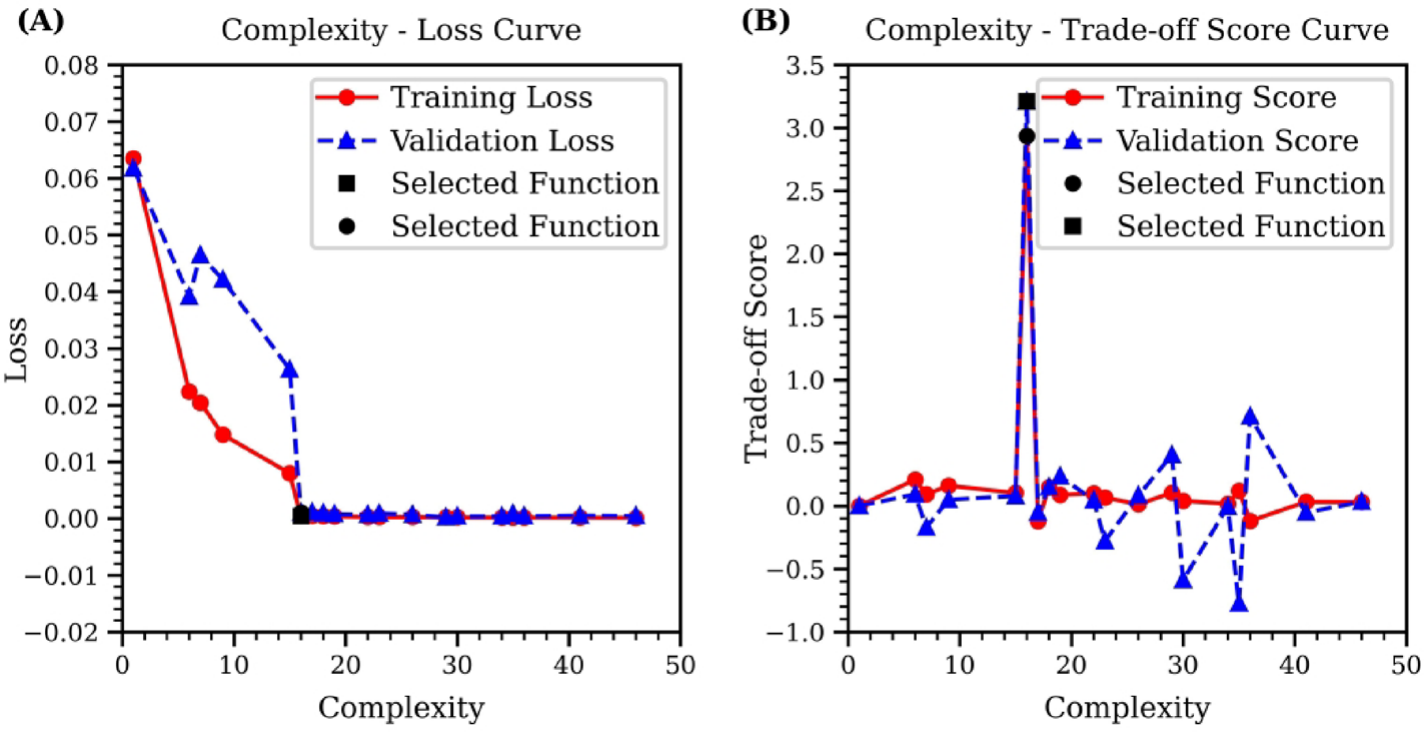
Complexity curves of symbolic regression using high-temperature GNN data. (**A**) illustrates the Complexity-Loss curve, highlighting the optimal equation at the turning point of minimal loss. (**B**) displays the Complexity-Trade-off Score curve, where the highest trade-off score indicates the selected equation that best balances simplicity with predictive accuracy.

The results of the symbolic regression using the high-temperature GNN data are presented in Fig. 8. Similar to Figs. 7 and 8A displays the Complexity-Loss curve, while Fig. 8B shows the Complexity-Trade-off Score curve. The optimal function is identified as the one with the highest training and test trade-off scores, signifying that it has achieved a balance between loss and complexity. The selected function is detailed below:

5 $$\:y=\:0.00945787\:-4.105327/({x}^{6}+0.8596078x+{0.8596078/(x}^{6}-x))$$

The function (5) suggests that the symbolic regression has identified a complex relationship in the high-temperature dataset. It correctly captured the $\:{1/x}^{6}$ attraction term but has replaced the $\:{1/x}^{12}$ repulsive term with a complex expression. This outcome reflects limitations in this dataset, which may arise not only from the elevated simulation temperature but also from insufficient sampling of atom pair distances. At high temperatures above the glass transition, the system naturally contains more atom pairs with distances greater than the equilibrium value, emphasizing the attractive region of the potential. However, the lack of sufficient atom pairs at shorter distances—where repulsive interactions dominate—makes it difficult for symbolic regression to learn the correct repulsive scaling. This imbalance leads the model to overfit to a narrow portion of the energy landscape. Therefore, to improve the robustness and accuracy of the learned potential, it is crucial to ensure that the dataset spans a more complete range of pair distances, which may require more targeted sampling strategies beyond simply adjusting system temperature.

## Discussion

In this study, we have introduced a novel approach that combines GNN with symbolic regression to reconstruct pair potential energy functions from simulation datasets. We selected the normalized Lennard-Jones system as a test case to validate our proposed methodology due to its simplicity and well-understood properties. This system, which yields the total potential energy as the sum of all pairwise potential energies based on only the interatomic distance, *x*, serves as a straightforward and comprehensible benchmark. In addition, the analytic form of the Lennard-Jones function includes terms to the sixth and twelfth power of *x*, which is complex enough to challenge the ability of symbolic regression models to capture the correct relationships between atom structure with potential energy. Given the defined analytical functional form, we can directly compare the function derived by our method with the ground truth function without needing additional validation steps. This case effectively showcases the capability of our approach to generate interpretable and precise analytical functions through the integration of GNN and Symbolic Regression.

Initially, a comprehensive MD dataset was gathered through MD simulations to train and test the GNN model. During the simulation process, datapoints were collected across various temperatures with sufficient time intervals to ensure that all atoms moved an adequate distance, thus losing any spatial memory from the previously collected datapoint. This approach ensured that the dataset was both comprehensive and uncorrelated. Each data point in the dataset contains the positions of each atom and the corresponding total potential energy. To enhance structural diversity, each simulation was initialized with a different configuration. The trained GNN demonstrated a robust ability to predict the system potential energy based on the atom positions and to accurately capture the value of pair potential energy via edge output features. Subsequently, the test data collected from MD simulation were input into the GNN model, and the pairwise distances and corresponding edge model output values from this test MD data were compiled to form the GNN dataset. For the symbolic regression model, we have investigated how the dataset is collected during molecular dynamics simulation influences the outcome of symbolic regression. The system temperature during simulation affects the average atomic distance. It was observed that the Symbolic Regression model identifies more optimal functions using a dataset accumulated at system temperatures under glass transition temperature due to their more extensive numerical range. This outcome is attributed to better coverage of short-range, high-curvature energy regions for this dataset, which are crucial for learning the steep repulsive portion of the potential. This technique has proven effective in extracting the normalized Lennard-Jones function from molecular dynamics simulation datasets, suggesting its applicability to more complex systems.

Although the reference system considered here is relatively simple, it effectively demonstrates how our method bridges the gap between black-box machine learning models and traditional analytical physics. Unlike purely ML-based potentials, such as deep neural networks or graph-based models^25,26^, which offer high accuracy but lack interpretability, our approach yields explicit analytical expressions for the potential energy function. These expressions are human-readable, physically meaningful, and can be directly integrated into classical MD simulations without additional runtime cost. While both our method and ML-based potentials fall under the broader category of data-driven approaches, our symbolic regression framework provides a distinct balance: it extracts interpretable functions from system-level energy data without requiring labeled pairwise energy terms. This capacity to derive pair potentials from system-level data highlights the broader applicability of our approach, whether in defining parameters for known potential forms or discovering new functional forms in complex systems where many-body effects and local coordination environments are difficult to model using traditional techniques.

The accuracy of this method depends strongly on the quality of the dataset, which must be free from spatial correlations and must span a wide range of atomic pair distances to avoid overfitting. To improve dataset quality, several strategies can be employed. First, systematically varying the quenching conditions, such as using different cooling rates sampled logarithmically, would allow the dataset to better capture structural variations associated with glass formation dynamics. Second, modifying system size or using different periodic box shapes could help test the sensitivity of the learned potential to finite-size effects and ensure that the model generalizes across simulation scales. Incorporating these strategies would increase atomic diversity and improve the robustness and transferability of the GNN-derived representations.

Our method also shows strong potential for use in ab-initio simulation settings, where only system-level potential energies are available and pairwise decomposition is not accessible. In such scenarios, our approach can extract interpretable pair potentials from existing data and identify analytical forms consistent with atomic interactions. This opens up new possibilities for computational material science, especially in materials where experimental or theoretical parameterizations are incomplete. For example, in disordered oxide glasses such as SiO₂ and borosilicate, researchers face competing two-body potential forms and parameter sets, with models such as BKS and SHIK yielding similar structures but differing in thermodynamic predictions^27,28^. This ambiguity arises from the inherent complexity of disordered networks, which are sensitive to coordination changes and local geometry. Our approach, which derives interpretable pair potentials from system-level energy data, offers a data-driven solution to this challenge. Moreover, in systems such as metallic glasses, where two-body interactions are fundamentally insufficient due to delocalized, many-body metallic bonding^29^, our method provides a potential direction for future extension to multi-body potential extraction, enabling more accurate modeling of complex materials.

However, we acknowledge several limitations when generalizing this method to complex systems, particularly those involving many-body effects, and note that trade-offs between accuracy and computational efficiency must be carefully considered. First, the edge attribute used in our GNN is defined as the Cartesian coordinate difference between atom pairs within a cutoff distance, with edge values beyond the cutoff set to zero. While this representation retains directionality and works well in the Lennard-Jones system, it lacks rotational invariance and introduces discontinuities at the cutoff boundary. This can reduce generalizability and may lead to instability in predictions near the cutoff threshold. Furthermore, a hard cutoff does not reflect the smooth decay of physical interactions in real materials. To address this, future work could explore rotationally invariant descriptors such as pairwise distances or angular features and adopt smooth cutoff functions (e.g., cosine or exponential decay) to maintain continuity. We note, however, that adopting a soft cutoff or higher-order descriptors would increase the complexity of the learned potential and could make it more challenging for symbolic regression to recover compact, interpretable expressions.

Second, the current GNN architecture and symbolic regression step assume that the system energy can be decomposed into a sum of two-body terms. While this assumption holds for the Lennard-Jones system, it may not generalize to systems with significant many-body effects. Nevertheless, the message-passing structure of the GNN inherently captures geometric information such as bond angles and local structural motifs, offering a basis for representing many-body interactions. To support more complex systems, additional node features—such as atomic charge, electronegativity, or valence—can be incorporated to better distinguish atomic environments. Moreover, symbolic regression can be extended beyond pairwise distance dependence to include higher-order GNN outputs such as node-level or edge-level embeddings that encode local many-body information. These symbolic components can then be combined into a composite energy function. This strategy would enhance the expressiveness of the model while maintaining interpretability.

In addition, we recognize that extending the method to many-body systems involves a fundamental trade-off between accuracy and computational efficiency. Compared to classical many-body potentials such as EAM or Stillinger-Weber^27^, which are manually parameterized and computationally efficient but often limited in flexibility and transferability, our framework can automatically discover functional forms from data with minimal prior assumptions. This adaptivity provides greater modeling power but may come with increased symbolic complexity. Based on the earlier discussion, applying our method to more complex many-body systems would require enhancements in both the GNN architecture and the symbolic regression step. For instance, capturing many-body effects would involve using richer structural features such as angular terms, smooth (soft) cutoffs, and rotationally invariant descriptors. These additions, while improving model fidelity, would significantly increase the complexity of the GNN output space and make the symbolic regression task more computationally demanding. In some cases, it may be necessary to fit multiple functional forms to different energy components (e.g., pairwise, angular, or higher-order contributions), further raising the symbolic regression cost. In comparison, deep learning-based potentials such as SchNet^25^ or MACE^26^ naturally encode many-body interactions and achieve high accuracy across diverse systems. However, these models require substantial computational resources during inference and training, particularly in large-scale MD simulations, and lack direct interpretability. Our approach, on the other hand, incurs greater cost during model development—especially when targeting many-body systems—but once symbolic regression produces a final analytical expression, it can be used in MD simulations with negligible runtime cost, akin to classical force fields. This one-time cost is amortized over all future simulations using the derived potential. In future work, we aim to quantify these trade-offs more rigorously by benchmarking symbolic regression-derived many-body potentials against classical and ML-based models in terms of both computational efficiency and accuracy.

Despite current limitations, our work establishes a transparent and extensible framework that provides a strong foundation for future generalization to more realistic systems involving many-body interactions. This approach not only demonstrates broad potential for molecular dynamics applications but also contributes to improved physical insight and predictive accuracy in materials modeling. We believe this framework opens new possibilities for integrating interpretability, efficiency, and accuracy in atomistic simulations.

## Conclusions

We finish the paper with our most important conclusions.

- The assembled GNN model demonstrates an unexpected ability to capture the correct pair potential energy directly from system energy based solely on the atomic structure. This opens up the potential to apply this method to cases where the pair potential energy is unknown and only the system energy is available, such as in ab-initio simulation scenarios.
- Unlike black box ML force fields, our method determines interatomic potential energy from system energy and generates functions that can be used in MD simulations without any additional computational cost. These functions are easily interpretable, facilitating further study.
- Interestingly, the function generated by symbolic regression with datasets collected under high simulation temperatures fails to capture the true Lennard-Jones interactions. This failure is attributed to the lack of atom pairs with low interaction distances in the high-temperature data. By contrast, symbolic regression analysis successfully generated the correct function based on low-temperature data.
- The quality of the dataset significantly impacts the accuracy of the functions generated by symbolic regression. It is important to have a diverse range of atomic distances in the dataset for accurate function generation.
- This method provides a potentially promising foundation for future extension to more complex systems with unknown potential energy functions or parameters, and may serve as a useful tool for advancing computational materials science as its capabilities are further developed.

## Electronic supplementary material

Below is the link to the electronic supplementary material.

Supplementary Material 1

## Data Availability

The data and code are available for noncommercial use at GitHub: https://github.com/ruoxia-c/GNNwithSymbolicRegression.
