# Supplementary material for: Using graph neural network and symbolic regression to model disordered systems
Source: Sci Rep. 2025 Jul 1;15:22122. doi: 10.1038/s41598-025-05205-8 (PMC12218424; doi:10.1038/s41598-025-05205-8)
Supplement: Supplementary file 1 — Supplementary Material 1 [file 41598_2025_5205_MOESM1_ESM.pdf]

Supplementary Information for: Using Graph Neural Network and Symbolic Regression to Model Disordered Systems

Ruoxia Chen*[a], Mathieu Bauchy[b], Wei Wang[c], Yizhou Sun[d], Xiaojie Tao[e], Jaime Marian*[f]

a. Physics of AmoRphous and Inorganic Solids Laboratory (PARISlab), Department of Civil and Environmental Engineering, University of California, Los Angeles, CA 90095, USA, ruoxia@g.ucla.edu
b. Physics of AmoRphous and Inorganic Solids Laboratory (PARISlab), Department of Civil and Environmental Engineering, University of California, Los Angeles, CA 90095, USA, bauchy@ucla.edu
c. Department of Computer Science, University of California, Los Angeles, CA 90095, USA, weiwang@cs.ucla.edu
d. Department of Computer Science, University of California, Los Angeles, CA 90095, USA, yzsun@cs.ucla.edu
e. Department of Mechanical and Aerospace Engineering, University of California Los Angeles, Los Angeles, CA 90095, USA, xiaojietao@g.ucla.edu
f. Department of Materials Science and Engineering, University of California, Los Angeles, CA 90095, USA, jmarian@g.ucla.edu

*. Corresponding authors:
Ruoxia Chen: ruoxia@g.ucla.edu
Jaime Marian: jmarian@g.ucla.edu

S1. GNN Training Result

S1.1 Training and Validation Loss Curves

To prevent overfitting and improve generalization, early stopping was implemented with a patience of 200 epochs based on validation loss. If no improvement in the validation RMSE was observed for 200 consecutive epochs, training was terminated early. The model parameters from the epoch with the lowest validation loss were retained for final evaluation.

To evaluate the convergence behavior and assess potential overfitting, we tracked both training and validation root mean squared error (RMSE) between the predicted global feature and the target system potential energy from the MD dataset throughout the training process. Figure S1 shows the training and validation loss curves over 1,200 epochs. Note that the plot begins from iteration 10 to better visualize loss curve trends in the later training stages, where more informative patterns emerge. As shown, both training and validation RMSE decrease rapidly in the early stages and continue to decline steadily before plateauing after approximately 700 epochs. The two curves remain closely aligned throughout the entire training process, with no observable divergence. This

consistent behavior indicates that the model maintains good generalization and does not suffer from overfitting. The final model was selected based on the epoch with the lowest validation RMSE using the early stopping criterion. For this run, the final training and test RMSE were 2.2069 and 2.2654, respectively, corresponding to 0.0172 and 0.0177 RMSE per atom.

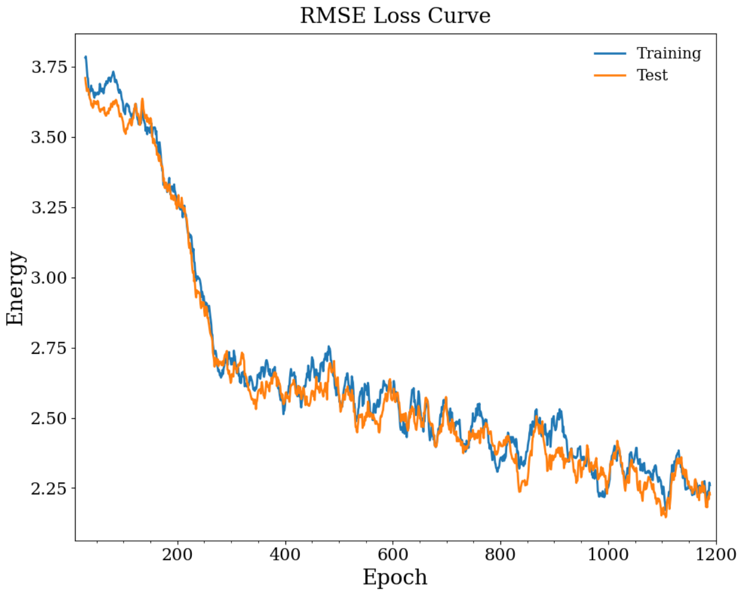

Figure S1. Training and validation RMSE per atom versus epoch for the GNN model.

S1.2 Uncertainty Estimation from Multiple Runs

To quantify the uncertainty in model performance, we trained the GNN model across five independent runs using different random seeds. For each run, we evaluated both the total RMSE and the per-atom RMSE on the training and test datasets. The results are summarized in Table S1.

Run	Train RMSE Total	Train RMSE per Atom	Test RMSE Total	Test RMSE per Atom
**1**	2.206854	0.017241	2.265409	0.017699
**2**	5.108926	0.039913	4.897506	0.038262
**3**	3.982509	0.031113	5.079448	0.039683
**4**	3.296132	0.025751	3.669976	0.028672
**5**	2.011696	0.015716	2.025610	0.015825
**Mean**	3.321223	0.025947	3.587590	0.028028
**Standard Deviation**	1.283597	0.010028	1.426202	0.011142

Table S1. GNN Training Results Across 5 Runs

The results demonstrate a high level of consistency across independent runs. The mean test RMSE per atom is 0.028028, with a standard deviation of 0.011142, indicating low variability in prediction accuracy due to random initialization. This stability reflects the robustness of the GNN architecture in learning meaningful representations from the system-level energy data.

Importantly, the predicted test RMSE per atom is small compared to the average potential energy per atom in the MD dataset, which is –2.0542. This corresponds to a relative error of approximately 1.36%, confirming that the model accurately captures the system-level potential energy landscape.

Moreover, the difference between training and test RMSE across all runs remains small (on average within 0.8% relative difference), suggesting that the model does not exhibit signs of overfitting. This is further supported by the loss curves shown in Section S1.1, where both training and validation loss converge smoothly and plateau without divergence. Together, these results confirm that the GNN achieves strong generalization from training data to unseen configurations and provides reliable predictions for use in downstream symbolic regression.

S2. Effect of Data Sampling on Symbolic Regression Performance

To clarify the influence of data sampling on symbolic regression, we analyzed the distribution of high-energy (short-distance) pairwise interactions in both the low- and high-temperature training datasets generate from trained GNN model. Figure S2 shows the normalized energy distribution for energy values greater than zero, which primarily correspond to atomic pairs with interatomic distances shorter than the equilibrium bond length.

In a Lennard-Jones (LJ) system, the equilibrium bond distance corresponds to the potential energy minimum at approximately 1.12σ. In low-temperature systems (below the glass transition temperature), thermal motion is suppressed, and atoms tend to settle near equilibrium positions. As a result, atomic pairs more frequently occupy short-distance configurations, spending more time near the bottom of the potential well. This leads to a higher proportion of positive energy values in the dataset, as confirmed by the broader and more frequent distribution of such values in the low-temperature curve. In contrast, in high-temperature systems, atoms vibrate more freely and occupy a wider range of distances, but due to the nature of the LJ potential, this often leads to fewer interactions with short-range repulsion and positive energy values.

This distributional difference is critical for symbolic regression. Since the positive energy range corresponds to the repulsive, steep-gradient part of the potential curve, the absence of sufficient training data in this region can cause overfitting and poor generalization, as obversed in the high-temperature dataset. On the other hand, the low-temperature dataset provides better coverage of

this sensitive region, enabling symbolic regression to extract a more accurate and generalizable potential energy function.

We acknowledge that this effect may partially stem from a sampling issue. The coverage of the potential energy landscape, especially in regions with short-range repulsion, plays a crucial role in model performance. This highlights an important consideration in dataset construction: it is essential not only to sample a wide range of configurations but also to ensure a more uniform and representative distribution of energy values, particularly in physically significant regions of the potential.

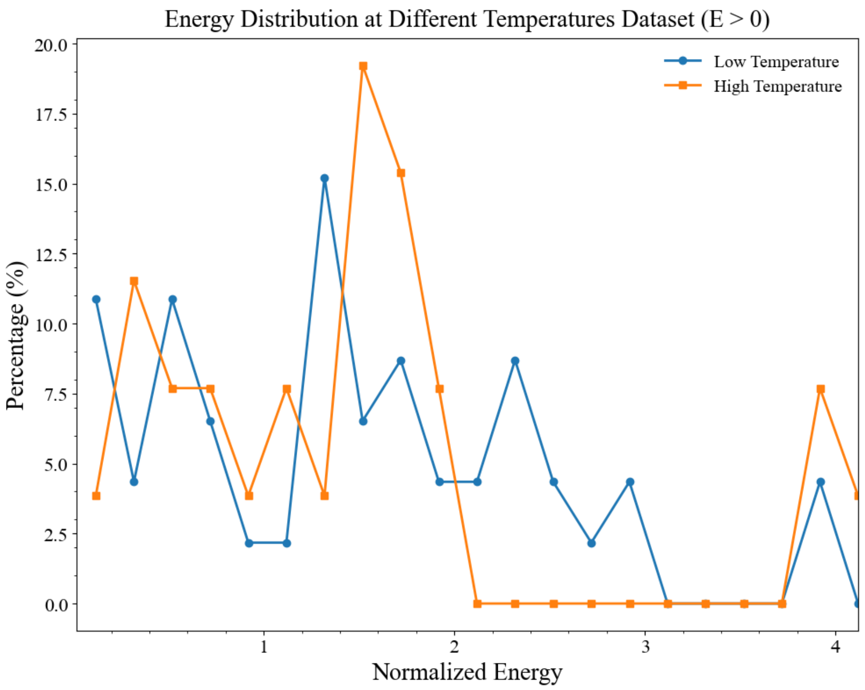

Figure S2. Percentage distribution of normalized pairwise energy values greater than zero in the low- and high-temperature GNN datasets.
